# The relative risk of second primary cancers in Austria’s western states: a retrospective cohort study

**DOI:** 10.1186/s12885-017-3683-9

**Published:** 2017-10-24

**Authors:** Oliver Preyer, Nicole Concin, Andreas Obermair, Hans Concin, Hanno Ulmer, Willi Oberaigner

**Affiliations:** 1 Agency for Preventive and Social Medicine, Bregenz, Vorarlberg Austria; 2 Department of Obstetrics and Gynaecology, Medical University of Innsbruck, Innsbruck, Austria; 3 Research Gynaecological Oncology, Queensland Centre for Gynaecological Cancer, Royal Brisbane and Women’s Hospital, 6th Floor Ned Hanlon Building, Brisbane, QLD Australia; 4 Department of Medical Statistics, Informatics and Health Economics, Medical University of Innsbruck, Innsbruck, Austria; 5 Department of Clinical Epidemiology of the Tyrolean State Hospitals Ltd, Cancer Registry of Tyrol, Tirolkliniken GmbH, Innsbruck, Austria; 6 Department of Public Health, Health Services Research and Health Technology Assessment, Institute of Public Health, Medical Decision Making and HTA, UMIT the Health & Life Sciences University, Hall in Tirol, Austria

**Keywords:** Relative risk, Second primary cancer, Austria, Retrospective, Cohort study

## Abstract

**Background:**

Cancer survivors are at risk of developing a second primary cancer (SPC) later in life because of persisting effects of genetic and behavioural risk factors, the long-term sequelae of chemotherapy, radiotherapy and the passage of time. This is the first study with Austrian data on an array of entities, estimating the risk of SPCs in a population-based study by calculating standardized incidence ratios (SIRs).

**Methods:**

This retrospective cohort study included all invasive incident cancer cases diagnosed within the years 1988 to 2005 being registered in the Tyrol and Vorarlberg Cancer Registries. Person years at risk (PYAR) were calculated from time of first diagnosis plus 2 months until the exit date, defined as the date of diagnosis of the SPC, date of death, or end of 2010, whichever came first. SIR for specific SPCs was calculated based on the risk of these patients for this specific cancer.

**Results:**

A total of 59,638 patients were diagnosed with cancer between 1988 and 2005 and 4949 SPCs were observed in 399,535 person-years of follow-up (median 5.7 years). Overall, neither males (SIR 0.90; 95% CI 0.86–0.93) nor females (SIR 1.00; 95% CI 0.96–1.05) had a significantly increased SIR of developing a SPC. The SIR for SPC decreased with age showing a SIR of 1.24 (95% CI 1.12–1.35) in the age group of 15–49 and a SIR of 0.85 (95% CI 0.82–0.89) in the age group of ≥ 65. If the site of the first primary cancer was head/neck/larynx cancer in males and females (SIR 1.88, 95% CI 1.67–2.11 and 1.74, 95% CI 1.30–2.28), cervix cancer in females (SIR 1.40, 95% CI 1.14–1.70), bladder cancer in males (SIR 1.20, 95% CI 1.07–1.34), kidney cancer in males and females (SIR 1.22, 95% 1.04–1.42 and 1.29, 95% CI 1.03–1.59), thyroid gland cancer in females (SIR 1.40, 95% CI 1.11–1.75), patients showed elevated SIR, developing a SPC.

**Conclusions:**

Survivors of head & neck, bladder/kidney, thyroid cancer and younger patients show elevated SIRs, developing a SPC. This has possible implications for surveillance strategies.

**Electronic supplementary material:**

The online version of this article (10.1186/s12885-017-3683-9) contains supplementary material, which is available to authorized users.

## Background

Multiple primary cancers are defined as the occurrence of two or more primary cancers, where each cancer originates in a separate primary site and is not an extension, recurrence or metastasis of the other cancer [1]. Two or more primary carcinomas can coexist at the time of diagnosis (synchronous) or develop later (metachronous), sometimes years after the first primary.

The criteria for defining second primary cancers have evolved over time and sometimes differ among studies. Using rules, registries are able to discriminate between new cancer cases and metastases of an existing malignancy. For international comparisons a unified definition of second primary cancers would be helpful. In our study we strictly followed the definition of the International Association of Cancer Registries (IACR) and the International Agency for Research on Cancer (IARC) as it is used widely [1]. The IARC/IACR rules are more exclusive; only one tumour is registered for an organ, irrespective of time, unless there are histological differences [1].

The Surveillance Epidemiology and End Results (SEER) Program takes account of histology, site, laterality and time since initial diagnosis to identify multiple primary cancers. SEER rules are mainly used by North American cancer registries. SEER currently collects and publishes cancer incidence and survival data from population-based cancer registries covering approximately 28% of the US population [2].

Up to 10% of cancer patients acquire multiple primary cancers at separate organ sites in the 10 years following the diagnosis of the first primary cancer [3]. In the SEER registry cancer survivors had a 14% higher risk of developing a new malignancy than the general population [4]. In Austria approximately 38,000 people are diagnosed with cancer annually, the number of prevalent cancer cases is 306,500, which represents about 4% of the population [5]. As demonstrated by the most recent publication of the EUROCARE-5 Working Group [6], Austria’s survival rates are high for the most frequent cancer sites [6].

The western provinces Vorarlberg and Tyrol have been covered by cancer registries since 1993 and 1988, respectively. The data of these two registries have reached a degree of completeness and data quality to be accepted for publication in Cancer Incidence in Five Continents [7].

There is increased surveillance in cancer survivors that could be a potential bias towards increased standardized incidence ratios (SIRs) even in the absence of an increase in the underlying risk.

The present retrospective cohort study investigated the relative risk of second primary cancers sites in Austria’s most western federal states firstly for all main primary cancer sites with a sufficient number of second primary cancer cases and secondly for all primary cancer sites aggregated in a single group. We estimated the relative risk of secondary primary cancers in a population-based study in western Austria by calculating SIRs. SIR is the established estimator in calculating the relative risk for multiple primary cancers (MPC) in population based cancer registries [8–11]. Our study is an examination of over 59.000 survivors of incident primary cancer with almost 400.000 person-years of follow-up. As this is the first study with Austrian data on an array of entities, we decided to publish these data based on a good quality population-based cancer registry, to support oncologists, epidemiologists and public health experts in their decision making process. Simultaneously we provide an useful addition to existing literature on second cancer risk in cancer survivors.

## Methods

This is a retrospective cohort study. In 2010 the cancer registries of the Austrian states of Tyrol and Vorarlberg covered a population of 707,485 and 369,453 respectively. Data of both registries are published in Cancer Incidence in Five Continents [7]. We included all invasive incident cancer cases diagnosed between 1988 and 2005 in adult patients (age ≥ 15 years). We excluded non-melanoma skin cancers, death certificate only (DCO) cases (below 4% for the whole observational period and below 2% since 1995), cases with a survival of less than 2 months and cases with a second primary cancer within 2 months after diagnosis, ending up in a total of 59,638 patients.

Patients were followed in a passive way by performing a probabilistic record linkage between incidence data and the official mortality data provided by Statistics Austria [12, 13]. Life status could not be assessed in 17 cases. These cases were excluded from analysis. The cohort was followed up until the end of 2010, thus allowing a follow-up of at least 5 years. The exit date was the date of diagnosis of the second primary cancer, date of death, or end of 2010, whichever came first.

Events were defined as first new primary cancer occurring at least 2 months after the diagnosis of the first primary cancer. Second primary cancers found at the same time of diagnosis of the first primary cancer (synchronous cancers) or occurring within 2 months after the first primary cancer diagnoses were excluded. Due to methodological matters third or subsequent primary cancers were excluded from this analysis. Additionally the risk for third and subsequent primary cancers is substantially lower (<1%) than that of second primary cancers in our group of patients.

Multiple cancers were assessed according to IARC definitions [14]. All cancer diagnoses were coded according to International Classification of Diseases for Oncology (ICD-O) Version 3 (cases diagnosed before 2001 have been reclassified), ICD-O was transformed to ICD10 applying a tool provided by IARC [15]. Cancer diagnosis was analysed based on ICD10-Codes, some codes have been aggregated according to topography (for example head/neck/larynx sites and colon/rectum). Tables were configured in the order of ICD10-codes. All cancer sites with at least 40 s primary cancer cases were analysed as a separate group.

Person years at risk (PYAR) were calculated from time of first diagnosis plus 2 months to the exit date defined above, as we did not count second primary cancers in the time slot of 2 months after the first primary cancer. The expected number of second primary cancers was calculated stratified by sex, age at time of first diagnosis grouped in five-year intervals and years of follow up grouped in five-year intervals as sum of PYAR multiplied by the incidence in the general population of Tyrol and Vorarlberg aggregated in one group in the respective stratum.

The SIRs of second primary cancer for the total of primary cancers were calculated as well as for specific primary cancers. SIRs were defined as the quotient of observed by expected cases and can be interpreted as risk of a cancer patient to develop a second primary cancer relative to the incidence rate in the general population. SIR for specific second primary cancers was calculated based on the risk of these patients for this specific cancer [16].

SIR was calculated for all second primary cancer sites aggregated in one group (in this situation the underlying cancer risk was defined by total cancer risk) and for specific second primary cancer sites (in that case the underlying cancer risk was defined as the cancer risk for that specific cancer site). In contrast, Table 3 and the supporting material (Additional file 1) show the risk for second primary cancer for the main cancer sites only (i.e. only those with 40 or more second primary cancer cases).

Analyses were performed with Stata Version 11.2 (Stata V11.2: Stata Statistical Software: Release 11. College Station, Tx: StataCorp LP; 2009). All patient data were non-identifiable.

## Results

### Baseline characteristics and distribution of first primary cancers by cancer type

Table 1 shows the baseline characteristics of the analysed study cohort of 59,638 patients diagnosed with cancer between 1988 and 2005. The most common first primary cancer entities of all patients were prostate cancer (16.9%) followed by breast cancer (14.8%) and colon/rectum cancer (12%). 4949 s primary cancers were observed over 399,535 person-years of follow-up. The median follow-up was 5.7 years (interquartile range 1.4–10.3).

**Table 1:** Characteristics of the study cohort

Study cohort	First primary cancers		Second primary cancers	
	Number of cases	%	Number of cases	%
Total	59,638	100	4949	100
Sex				
Males	31,215	52.3	3036	61.3
Females	28,423	47.7	1913	38.7
Age at first diagnosis				
15–24	656	1.1	8	0.2
25–49	9143	15.3	439	8.9
50–64	19,119	32.1	1784	36.0
65–79	23,424	39.3	2356	47.6
80+	7296	12.2	362	7.3
Period of first diagnosis				
1988–1990	7764	13.0	706	14.3
1991–1995	15,140	25.4	1487	30.0
1996–2000	16,837	28.2	1526	30.8
2001–2005	19,897	33.4	1230	24.9
Follow-up interval				
2 months to less than 1 year	8851	14.8	546	11.0
1 year to less than 5 years	13,279	22.3	1928	39.0
5 years to less than 10 years	17,563	29.4	1522	30.8
10 years or longer	19,945	33.4	953	19.3

The distribution of first primary cancers by cancer types is shown in Table 2.

**Table 2:** Distribution of first primary cancers by cancer type

First primary cancers	Number of cases	%
Head/neck/larynx	2322	3.9
Stomach	2974	5.0
Colon/Rectum	7138	12.0
Lung	5134	8.6
Breast	8833	14.8
Cervix	1290	2.2
Endometrium	1768	3.0
Ovary	1310	2.2
Prostate	10,103	16.9
Bladder	2420	4.1
Kidney	1866	3.1
Thyroid gland	1088	1.8
NHL high/low	1070	1.8
CLL/ALL	573	1.0
Other	8704	14.6

*NHL* non-Hodgkin Lymphoma, *CLL* chronic lymphatic leukaemia, *ALL* acute lymphatic leukaemia

### Distribution of gender, site and time of occurrence of second primary cancers

In our data we did not observe relevant differences (absolute difference ≤ 0.2) of SIR between females and males in each entity except for lung cancer 0.90 (95% CI 0.75–1.06) for males versus 1.58 (95% CI 1.19–2.05) for females. Full details are shown in Table 3. About one in 10 s primary cancers (11%) was diagnosed within 1 year after the first diagnosis, 39% of the second primary cancers were diagnosed within one to 5 years of the first diagnosis and 50% after 5 years. Prostate cancer accounts for about 1/3 of all cancer cases in males in our cohort.

**Table 3:** SIRs of second primary cancer by type of first primary cancer and sex

Sex	Males		Females	
First primary cancer	Obs.	SIR (95% CI)	Obs.	SIR (95% CI)
Head/Neck/Larynx	281	**1.88 (1.67–2.11)**	53	**1.74 (1.30–2.28)**
Stomach	111	0.94 (0.77–1.13)	57	0.83 (0.63–1.07)
Colon/Rectum	381	0.91 (0.82–1.00)	233	0.89 (0.78–1.01)
Lung	130	0.90 (0.75–1.06)	56	**1.58 (1.19–2.05)**
Melanoma	133	0.93 (0.78–1.10)	107	0.94 (0.77–1.14)
Breast			580	***0.82 (0.75–0.89)***
Cervix			102	**1.40 (1.14–1.70)**
Endometrium			177	1.08 (0.92–1.25)
Ovary			72	1.09 (0.85–1.37)
Prostate	1117	***0.68 (0.64–0.72)***		
Bladder	300	**1.20 (1.07–1.34)**	61	1.13 (0.86–1.45)
Kidney	162	**1.22 (1.04–1.42)**	84	**1.29 (1.03–1.59)**
Thyroid gland	40	1.23 (0.88–1.67)	79	**1.40 (1.11–1.75)**
NHL high/low	68	1.20 (0.93–1.53)	43	1.23 (0.89–1.66)
CLL/ALL	34	0.93 (0.64–1.30)	22	1.11 (0.69–1.67)
All cancers combined	3036	***0.90 (0.86–0.93)***	1913	1.00 (0.96–1.05)
All cancers except Prostate	1919	**1.10 (1.05–1.15)**		

*Obs.* Observed number of second primary cancers, *SIR* standardized incidence ratio, *CI* confidence interval, *NHL* non-Hodgkin Lymphoma, *CLL* chronic lymphatic leukaemia, *ALL* acute lymphatic leukaemia

SIRs shown in normal bold font indicate significantly increased risk, SIRs shown in bold italics indicate significantly decreased risk

### Age at diagnosis of the first primary cancer

The SIR for second primary cancer decreased with age showing a SIR of 1.24 (95% CI 1.12–1.35) in the age group of 15–49 years and a SIR of 0.85 (95% CI 0.82–0.89) in the age group of ≥65 years. The same pattern was seen in most cancer sites (for details see Table 4).

**Table 4:** SIR of second primary cancer by type of first primary cancer and age group at first diagnosis

	Age group at first diagnosis					
	15–49 years		50–64 years		65 years and over	
First primary cancer	Obs.	SIR (95% CI)	Obs.	SIR (95% CI)	Obs.	SIR (95% CI)
Head/Neck/Larynx	42	**2.35 (1.69–3.17)**	189	**2.21 (1.91–2.55)**	103	**1.34 (1.10–1.63)**
Stomach	8	1.14 (0.49–2.25)	43	0.92 (0.66–1.24)	117	0.88 (0.72–1.05)
Colon/Rectum	29	1.17 (0.78–1.68)	200	1.03 (0.89–1.18)	385	***0.83 (0.75–0.92)***
Lung	13	1.31 (0.70–2.25)	83	1.13 (0.90–1.40)	90	0.93 (0.74–1.14)
Melanoma	54	1.18 (0.89–1.54)	88	0.89 (0.71–1.10)	98	0.88 (0.71–1.07)
Breast	70	0.79 (0.62–1.00)	227	0.89 (0.78–1.02)	283	***0.77 (0.68–0.87)***
Cervix	43	**1.56 (1.13–2.11)**	33	1.28 (0.88–1.80)	26	1.32 (0.86–1.93)
Endometrium	11	1.21 (0.60–2.16)	67	1.06 (0.82–1.35)	99	1.07 (0.87–1.31)
Ovary	15	1.72 (0.96–2.83)	26	1.09 (0.71–1.59)	31	0.93 (0.63–1.31)
Prostate	15	**1.10 (0.62–1.81)**	326	***0.66 (0.59–0.74)***	776	***0.68 (0.63–0.73)***
Bladder	15	1.65 (0.93–2.73)	126	**1.41 (1.18–1.68)**	220	1.07 (0.93–1.22)
Kidney	21	1.54 (0.96–2.36)	106	**1.35 (1.10–1.63)**	119	1.12 (0.93–1.34)
Thyroid gland	23	1.05 (0.66–1.57)	53	**1.49 (1.12–1.95)**	43	1.37 (0.99–1.84)
NHL high/low	10	1.24 (0.59–2.27)	45	1.29 (0.94–1.73)	56	1.15 (0.87–1.50)
CLL/ALL	3	1.86 (0.38–5.44)	14	0.71 (0.39–1.19)	39	1.11 (0.79–1.52)
All cancers combined	447	**1.24 (1.12–1.36)**	1784	1.02 (0.97–1.07)	2718	***0.85 (0.82–0.89)***

*Obs.* Observed number of second primary cancers, *SIR* standardized incidence ratio, *CI* confidence interval, *NHL* non-Hodgkin Lymphoma, *CLL* chronic lymphatic leukaemia, *ALL* acute lymphatic leukaemia

SIRs shown in normal bold fond indicate significantly increased risk; SIRs shown in bold italics indicate significantly decreased risk

### SIR of second primary cancer by type of first primary cancer and sex

SIR of all cancers combined except prostate cancer was 1.00 (95% CI 0.96–1.05) for females and significantly decreased 0.90 (95% CI 0.86–0.93) in men. After exclusion of prostate cancer SIR was significantly increased at 1.10 (95% CI 1.05–1.15). The SIR for second primary cancers varied substantially according to the type of first primary cancer. There was a significantly increased SIR for second primary cancers in men after head/neck/larynx cancer (SIR 1.88; 95% CI 1.67–2.11), kidney cancer (SIR 1.22; 95% CI 1.04–1.42) and bladder cancer (SIR 1.20; 95% CI 1.07–1.34), see Table 3. Amongst women there was a significantly increased SIR for second primary cancers after head/neck/larynx cancer (SIR 1.74; 95% CI 1.30–2.28), lung cancer (SIR 1.58; 95% CI 1.19–2.05), cervical cancer (SIR 1.40; 95% CI 1.14–1.70), thyroid cancer (SIR 1.40; 95% CI 1.11–1.75) and kidney cancer (SIR 1.29; 95% CI 1.03–1.59), see Table 3, while women after breast cancer (SIR 0.82; 95% CI 0.75–0.89) and men after prostate cancer (SIR 0.68; 95% CI 0.64–0.72) had a significantly decreased SIR of developing a second primary cancer. Figures showing the SIR of further entities can be found in the supporting material section (see Additional file 1).

## Discussion

In our study we analysed the SIR for second primary cancers for the main entities. We found no increased SIR except for cancer of the head & neck, bladder/kidney and thyroid and an increased SIR for younger patients.

### Role of prostate cancer

The incidence of prostate cancer more than doubled in Tyrol in 1993 and some 5 years later in Vorarlberg due to the introduction of PSA screening in men aged 45 to 79. Prostate cancer accounts for about 1/3 of all cancer cases in males in our cohort and therefore had a major impact on the estimates for all cancer sites combined.

Therefore we were interested in an estimate for all cancer sites combined, except prostate cancer. Analysing this, the SIR in males for all cancer sites except prostate was slightly increased (10%) and this increase was statistically significant.

When comparing our results with other study results this observation should be kept in mind because the mix of cancer sites varies in some extent between countries [9, 15]. Our data with a reduced SIR for second primary cancers in patients with prostate cancer is in accordance to data published by Coyte et al. [8].

An inclusion of prostate cancer may alter the results due to radiation therapy. In Austria prostate-specific antigen (PSA) screening allows prostate cancer to be detected in a very early stage, achieving a very good prognosis. The underlying aetiology of developing a second primary cancer after prostate cancer may be related to various factors, including treatment modality. More than 50% of the small intestine tumours were carcinoid malignancies, suggesting possible hormonal influences. An excess of pancreatic cancer may be due to pathogenic variants, which predisposes to both [17].

### Role of definition

The criteria for defining second primary cancers have evolved over time and sometimes differ among studies. Using rules, registries are able to discriminate between new cases and metastases of an existing malignancy. Definitions are critical when analysing the SIR for second primary cancer. Internationally, both the definition of the International Association of Cancer Registries (IACR) and the International Agency for Research on Cancer (IARC) [1] as well as the rules of the Surveillance Epidemiology and End Results (SEER) Program [2] definitions are widely used and some registries use their own definitions [8, 9].

The IARC rules are more exclusive. Irrespective of time only one tumour is registered for an organ, unless there are histological differences. In contrast, North American cancer registries use the SEER rules that take account of histology, site, laterality and time since initial diagnosis to identify multiple primary cancers [2].

Coyte et al. demonstrated the implication of these differing definitions**:** for an aggregation of 10 cancer sites in the Scottish study, applying the IARC definition led to a SIR of 0.86 and SEER definition to a SIR of 1.0 [8]. At least for the Scottish data, the absolute difference in SIR is at about 0.15. Our estimates for all cancer sites combined at SIR 1.00 (0.96–1.05) for females and SIR 0.90 (0.86–0.93) for males are in the range reported in different studies, namely from 1.08 to 1.3 [9, 18–20]. Therefore our estimates for all cancer sites combined are in line with published data by previous studies [8–10]. This observation was applicable for site specific results, e.g. increased SIR for primary cancer in the head & neck cancer, kidney, bladder and thyroid and reduced SIR in prostate. For female breast published results are inconsistent [7, 9] but the Scottish data from Coyte et al. are in line with our lowered SIR in women with breast cancer as a first primary [8].

### Role of age

Taking into account the variation of age specific incidence during the follow-up period in our method we found a consistent pattern of higher SIR for second primary cancer in younger patients (SIR 1.24 for patients aged below 50) and lower SIR in patients aged 65 and higher. Some of the results for SIR were significantly higher in the lowest age group (15–49 years). According to increased SIRs in head/neck/larynx, cervix and prostate cancer as well as in the all cancers combined group a more dense surveillance may be warranted. This observation is in line with results reported by previously published data [9] and has clinical implications such as more dense surveillance in younger cancer patients [9], but also the fact of their longer life expectancy. The risk compared to the age-matched general population was higher in survivors at younger ages, but within the survivor population, increasing age is still associated with increased cancer risk. In an other study it has been shown that adolescents and young adult cancer survivors, who survive more than 5 years have a higher relative risk of secondary malignant neoplasms compared with younger or older cancer survivors [21]. In addition we would like to notice that we calculated age adjusted SIRs.

### Genetic and behavioural risk factors

Possible reasons for an increased SIR for second primary cancers in cancer survivors are genetic and behavioural risk factors [11, 22, 23], treatment of the first primary cancer radiotherapy and chemotherapy, and more intense surveillance of prevalent cancer cases [24]. Lifestyle factors such as smoking (risk factor for head and neck/lung/bladder/kidney) and alcohol consumption are risk factors for a number of cancers. A lack of risk factor data in our cohort limits us to speculation regarding correlations. However, in our analysis the majority of primary cancer sites with increased SIR is nicotine-associated. Of course changing modifiable lifestyle factors like e.g. to quit smoking, will reduce the risk of second primary cancer but also risk of other diseases [25]. However, there is little knowledge on whether cancer survivors in fact are successful to change their habits and we have no data on this. There is some evidence that a cancer diagnosis in adults may have a positive influence on smoking and diet but a negative influence on exercise [26].

### Therapy as a risk

Radiation is a risk factor to neighbouring organs of the first primary cancer site. About half of all cancer patients receive radiotherapy at some stage of their disease in developed countries, and at least for some cancer sites like Hodgkin lymphoma, breast cancer, and some gynaecologic malignancies such as vulvar and endometrial cancer it has been shown that radiotherapy causes second primary cancers. These are lung, breast, stomach and thyroid cancer after Hodgkin lymphoma, contralateral breast, lung and oesophagus after breast cancer and leukaemia and any other secondary malignancy after vulvar or endometrial cancer, respectively [27–33].

SIRs and rates of secondary malignancies in high-risk populations have been influenced also by changes in chemotherapy protocols. Chemotherapy-sensitive tissues such as bone marrow, epithelial cells of the gastrointestinal tract and hair follicles are most likely to begin carcinogenesis, therefore the development of leukaemia and lymphoma as secondary hematologic cancers seem to be the greatest long-term risk to cancer survivors after chemotherapy [29–34].

Future effort of research might focus on the complex area of molecular mechanisms of second cancer development. In times of targeted therapies it becomes increasingly important to incorporate factors in our decision making process that might be able to predict the susceptibility of patients to both acute and chronic toxicity, including second primary cancers. This might offer opportunities to individualize therapy, to maximize therapeutic benefit and to minimize serious late toxicity [35].

### Surveillance matters

Increased surveillance after a first primary cancer leads to earlier detection of second primary cancers. For example routine use of ultrasound has been shown to dramatically increase thyroid cancer incidence on a population level and hence we would also expect a higher detection rate of thyroid cancer as second primary cancer [36].

### Why the risk of second primary cancers is so important

Age-specific mortality rates for chronic diseases are driven by changes in exposure to risk factors and by availability of screening systems and treatment. The risk of cancer after cancer in the overall population might be expected to rise because of persisting effects of genetic and behavioural risk factors, long-term side effects of chemotherapy and radiotherapy, and improved diagnostics [8]. Even if cancer incidence and survival rates remain stable, the number of cancer survivors in the United States will increase by 31%, to about 18.1 million, by 2020 [37]. Because of the aging of the U.S. population, the largest increase in cancer survivors over the next 10 years will be in the age group 65 and older. If new tools for cancer diagnosis, treatment, and follow-up continue to be more expensive, medical expenditures for cancer could reach as high as $207 billion [37]. Policies and programs modifying behavioural and environmental factors to reduce the burden of cancers are key [38].

These data also fit to different other high-income countries and therefore also for Austria. We are also confronted with an aging population. Due to a growing number of cancer survivors this becomes an increasing health concern also in Austria [39], as these patients may impact the overall quality of long-term care in this growing population, like elsewhere [40].

As more and more patients are surviving a cancer, preventing both recurrence and development of second primary cancers is a major goal of national health plans, as they are cost intensive in treatment and care [41]. Taking care of cancer survivors is becoming a challenge for health programs. Cancer survivors could benefit from a coordinated public health effort to support them, as they face numerous physical, psychological, social, spiritual, and financial issues throughout their diagnosis and treatment and the years thereafter. Support depends on the national health care system of the respective country. By preventing secondary diseases or recurrence of cancer and with it improving quality of life for each survivor, many of these issues could be successfully and more focally addressed. Patterns of secondary cancers as shown by our analysis would be helpful for deciding where to focus efforts. One focus of course must be primary prevention as it has already been shown to be the most effective way to fight cancer [42] and the reduction of exposure to key behavioural and environmental risk factors is key to prevent a substantial proportion of deaths form cancer [38].

### Strengths and limitations

The strength of our study is the high degree of data completeness of both registries over the full study period and the strict definition of second primary cancer. Furthermore, this is the first study with Austrian data on an array of entities. The limitations are the low population number which causes broader confidence intervals and limits the conclusions to draw especially for site specific results, the lack of registering key information on risk factors and more detailed information on treatment which in consequence does not allow us to analyse the impact of these factors on the risk of second primary cancer.

## Conclusions

The SIR of second primary cancer incidence in general might be expected to rise because of persisting effects of genetic and behavioural risk factors (e.g., smoking, lack of exercising, HPV infections), long-term side effects of chemotherapy and radiotherapy and better diagnostics. Our data show, that for all cancer sites combined, the SIR for of second primary cancer is increased only for men when we exclude prostate cancer. In our study the SIR for second primary cancer is consistently increased after first primary cancer of the head/neck, bladder/kidney as well as the thyroid and is also increased for younger patients, facts that can help focusing strategies for surveillance.

## Additional file

Additional file 1: SIRs of second primary cancer by type of first primary cancer and sex. Standard incidence ratios of all second primary cancers analysed are provided by type of first primary cancer and sex. (DOC 58 kb)

## Abbreviations

ALL: Acute lymphatic leukaemia
CLL: Chronic lymphatic leukaemia
DCO: Death certificate only
IACR: International Association of Cancer Registries
IARC: International Agency for Research on Cancer
ICD: International Classification of Diseases
ICD-O: International Classification of Diseases for Oncology
MPC: Multiple primary cancer
NHL: Non-Hodgkin Lymphoma
Obs.: Observations
PSA: Prostate-specific antigen
PYAR: Person years at risk
SIR: Standardized incidence ratio
SPC: Second primary cancer
