# Supplementary material for: The relative risk of second primary cancers in Austria’s western states: a retrospective cohort study
Source: BMC Cancer. 2017 Oct 24;17:699. doi: 10.1186/s12885-017-3683-9 (PMC5655958; doi:10.1186/s12885-017-3683-9)
Supplement: Additional file 1: — SIRs of second primary cancer by type of first primary cancer and sex. Standard incidence ratios of all second primary cancers analysed are provided by type of first primary cancer and sex. (DOC 58 kb) [file 12885_2017_3683_MOESM1_ESM.doc]

**Additional file 1. SIRs of second primary cancer by type of first primary cancer and sex**
